# Aspulvinone O, a natural inhibitor of GOT1 suppresses pancreatic ductal adenocarcinoma cells growth by interfering glutamine metabolism

**DOI:** 10.1186/s12964-019-0425-4

**Published:** 2019-08-30

**Authors:** Weiguang Sun, Shanshan Luan, Changxing Qi, Qingyi Tong, Shan Yan, Hua Li, Yonghui Zhang

**Affiliations:** 1 Department of Pharmacology, School of Pharmacy, Tongji Medical College, Huazhong University of Science and Technology, Wuhan, 430030 China; 2 Wuya College of Innovation, Key Laboratory of Structure-Based Drug Design & Discovery, Ministry of Education, Shenyang Pharmaceutical University, Shenyang, 110016 China

**Keywords:** Aspulvinone O, Pancreatic ductal adenocarcinoma cells, GOT1 inhibitor, Glutamine metabolism

## Abstract

**Background:**

Distinctive from their normal counterparts, cancer cells exhibit unique metabolic dependencies on glutamine to fuel anabolic processes. Specifically, pancreatic ductal adenocarcinoma (PDAC) cells rely on an unconventional metabolic pathway catalyzed by aspartate transaminase 1 (GOT1) to rewire glutamine metabolism and support nicotinamide adenine dinucleotide phosphate (NADPH) production. Thus, the important role of GOT1 in energy metabolism and Reactive Oxygen Species (ROS) balance demonstrates that targeting GOT1 may serve as an important therapeutic target in PDAC.

**Methods:**

To assay the binding affinity between Aspulvinone O (AO) and GOT1 proteins, the virtual docking, microscale thermophoresis (MST), cellular thermal shift assay (CETSA) and drug affinity responsive target stability (DARTS) methods were employed. GOT1 was silenced in several PDAC cell lines. The level of OCR and ECR were assayed by seahorse. To evaluate the in vivo anti-tumor efficacy of AO, the xenograft model was built in CB17/scid mouse.

**Results:**

Screening of an in-house natural compound library identified the AO as a novel inhibitor of GOT1 and repressed glutamine metabolism, which sensitizes PDAC cells to oxidative stress and suppresses cell proliferation. Virtual docking analysis suggested that AO could bind to the active site of GOT1 and form obvious hydrophobic interaction with Trp141 together with hydrogen bonds with Thr110 and Ser256. Further in vitro validation, including MST, CETSA and DARTS, further demonstrated the specific combining capacity of AO. We also show that the selective inhibition of GOT1 by AO significantly reduces proliferation of PDAC in vitro and in vivo.

**Conclusions:**

Taken together, our findings identify AO as a potent bioactive inhibitor of GOT1 and a novel anti-tumour agent for PDAC therapy.

**Electronic supplementary material:**

The online version of this article (10.1186/s12964-019-0425-4) contains supplementary material, which is available to authorized users.

## Background

Altered metabolism is a key hallmark of cancer cells to fulfill their biosynthetic requirements [1]. Most cancers depend on a high rate of aerobic glycolysis, known as the Warburg effect, for their continued growth and proliferation [2]. This characteristic metabolic phenotype involves enhanced glucose uptake and flux into glycolysis, with simultaneously diminishing the glucose carbon flux that enters the tricarboxylic acid (TCA) cycle in the mitochondria, even in the presence of oxygen [3, 4]. Paradoxically, some cancer cell lines, including pancreatic cancer cells lines, breast adenocarcinomas, acute myelogenous leukaemia lines, glioblastoma multiform cells and small cell lung cancer lines, also exhibit addiction to glutamine (Gln) despite the fact that glutamine is a nonessential amino acid that can be synthesized from glucose [5, 6]. The 10-fold of Gln uptake exhibited by Gln-dependent cells indicates that its role is not solely a nitrogen donor for nucleotide and amino acid biosynthesis [7]. Subsequent studies revealed that Gln is the primary mitochondrial substrate and is required to maintain mitochondrial membrane potential and integrity as well as support of the NADPH production needed for redox control and macromolecular synthesis [8, 9].

The importance of Gln metabolism has inspired extensive investigations into the spectrum of Gln -dependent cancers and the mechanisms by which Gln fuels cancer metabolism [10]. In many cancer cells, Gln is delaminated to glutamate and further converted into alpha-ketoglutarate (a-KG), to fuel the TCA cycle as a carbon source for the biosynthesis of nucleotides, nonessential amino acids, and hexosamines, which is essential for oncogenic Kras-induced tumor growth [11, 12]. Recent investigations have demonstrated that pancreatic ductal adenocarcinoma (PDAC) cells which are resistant to conventional or targeted therapies showed profoundly sensitive to Gln deprivation [13, 14]. PDAC cells metabolize Gln in a manner that is different from canonical model sand that Gln derived aspartate (Asp) was transported into the cytoplasm, in which glutamic-oxaloacetic transaminase 1 (GOT1) catalyses the conversion of aspartate (Asp) and α-KG into oxaloacetate (OAA) and glutamate (Glu), afterward, successive reactions catalyzed by malate dehydrogenase 1 (MDH1) and malic enzyme 1 (ME1) converted OAA to pyruvate and produced NADPH. PDAC cells are dependent on these reactions to increase the NADPH/NADP ratio for the maintenance of the intracellular reactive oxygen species (ROS) balance [15]. GOT1 as an essential in situ metabolic target of OAA had been demonstrate that selective inhibition of GOT1 with specific siRNA species decreases the proliferation of cancer cells. Therefore, GOT1 represents a valid molecular target for the development of anti-neoplastic agents [16].

Natural products (NPs) have played a highly significant role in anticancer drug discovery and development [17, 18]. From the 1930s to 2014, approximately 60% of all small-molecule anticancer drugs approved were either NPs or their derivatives [19]. As the emphasis in cancer drug development has shifted from cytotoxic, non-specific chemotherapies to molecularly targeted, rationally designed drugs [20], NPs have been making a comeback by providing structurally interesting compounds and ‘targeted screening systems’ to search for valuable leads [21].

In this study, we report that the 3-[2,4-Dihydroxy-5-(3-methyl-2-buten-1-yl) phenyl]-4-hydroxy-5-[[4-hydroxy-3-(3-methyl-2-buten-1-yl) phenyl] methylene]-2(5H)-furanone, named as Aspulvinone O (AO), inhibited the growth of PDAC cells both in vitro and in vivo. Mechanically, AO suppressed GOT1 activity by competitive binding to the activation site thus inhibited Gln metabolism, resulting in apoptosis in cancer cells. Our work suggested that AO, a selective GOT1 inhibitor, has potential to be a novel agent for pancreatic cancer therapy.

## Methods

### Reagents

AO was isolated from the rice culture of *Aspergillus terreus* according to Supplementary Methods. Its structure was characterized by one-dimensional nuclear magnetic resonance (NMR) spectrometer (Additional file 1). AO (99% or higher purity) was dissolved in dimethylsulfoxide as a 50 mM stock solution and stored at − 20 °C. Antibodies against GOT1 and β-actin were purchased commercially from Santa Cruz Biotechnology; goat anti-mouse IgG and goat anti-rabbit IgG antibodies were purchased from Cell Signaling Technology. BenzyloxycarbonylVal-Ala-Asp fluoromethylketone (Z-VAD-FMK) was from Selleck; 20,70-dichlorodihydrofluorescein diacetate (DCFH-DA) was from Invitrogen, and other chemical reagents were from Sigma Aldrich.

### Cloning, expression and purification of GOT1

The genes were cloned into the pET26b vector (Novagen) containing a 6 His-tag coding region at the C-terminus of the insert. After verifying the recombinant plasmids by sequencing, the plasmids were used to transform *Escherichia coli* strain BL21 (DE3). The transformed cells were grown in LB medium at 37 °C to an OD_600_ (0.8–1.0) and induced with 0.4 mM isopropyl-D-thiogalactopyranoside (IPTG) at 293 K for 16 h. After harvested by centrifugation, the cells were re-suspended on ice in lysis buffer containing 20 mM Tris, pH 8.5, 200 mM NaCl, and 10 mM imidazole, followed by disruption on a French press. Cell debris was removed by centrifugation at 21,000 rpm for 30 min. The protein was bound to Ni-agarose affinity resin, washed with buffer containing 20 mM Tris, pH 8.5, 200 mM NaCl, and 10 mM imidazole, and eluted with buffer containing 20 mM Tris, pH 8.5, 250 mM NaCl, and 150 mM imidazole. The protein was further purified by anion-exchange chromatography using a linear gradient of 10 mM to 1 M NaCl and size exclusion chromatography at pH 8.5 in 200 mM NaCl.

### GOT1 inhibitory activity assay

The effect of AO on GOT1 was measured using purified human recombinant enzyme. In a 100 μL reaction, 4 mM Asp, 1 mM α-KG, 1 units/mL malate dehydrogenase, 1 mM NADH and 0.1 mg/mL human recombinant GOT1 were combined. Absorbance at 340 nm was measured using a 96-well plate reader to determine the maximum linear change of absorbance as a measurement of enzyme activity. GOT1 activity, in the presence of several concentrations of compounds was measured to determine in vitro inhibition. Enzyme activity was analyzed using Sigma PlotEnzyme Kinetics Module (Systat Software, Richmond, CA).

### Molecular docking

Crystal structures of human GOT1 (PDB code: 3II0) was obtained from the Protein Data Bank. The docking was operated by using ICM 3.8.2 modeling software on an Intel i7 4960 processor (MolSoft LLC, San Diego, CA). Ligand binding pocket residues were selected by using graphical tools in the ICM software, to create the boundaries of the docking search. In the docking calculation, potential energy maps of the receptor were calculated using default parameters. Compounds were inputted into ICM and an index file was created. Conformational sampling was based on the Monte Carlo procedure30, and finally the lowest-energy and the most favorable orientation of the ligand were selected.

### Microscale thermophoresis

Recombinant GOT1 was labelled with the Monolith NTTM Protein Labeling Kit RED (Cat # L001) according to the supplied labelling protocol. Labeled proteins were used at a concentration of 50 nM. Samples were diluted in a 20 mM HEPES (pH 7.4) and 0.5 (v/v) % Tween-20. We used 200 μM AO as the highest concentration for the serial dilution. After 10 min incubation at room temperature the samples were loaded into MonolithTM standard-treated capillaries and the thermophoresis was measured at 25 °C after 30 min incubation on a Monolith NT.115 instrument (NanoTemper Technologies, München, Germany). Laser power was set to 40% using 30 s on-time. The LED power was set to 100%. The dissociation constant (KD) values were fitted by using the NTAnalysis software (NanoTemper Technologies, München, Germany).

### Cell culture and viability assay

Several human cancer cell lines, including breast (MM231, MM453, HCC1806), colorectal (HCT116), pancreas (PANC-1, AsPC-1, SW1990), along with nonmalignant human hepatic cell line (HDPE6C7) were obtained from American Type Culture Collection (ATCC). Cells were cultured in Dulbecco’s modified Eagle’s medium (DMEM) or 1640 media supplemented with 10% (v/v) fetal bovine serum (FBS), 100 U/ml penicillin and 100 μg/mL streptomycin. Cells cultures were maintained at 37 °C in a humidified incubator of 5% CO_2_. Cell viability was assessed using a 3-(4,5-dimethylthiazol-2-yl)-2,5-diphenyltetrazoliumbromide (MTT) assay. Briefly, cells were plated at a density of 5 × 10^3^ cells per well in 96-well plates for 24 h. The medium was then removed, and cells were treated with either DMSO as a control or various concentrations (0.1–100 μM) of AO. After the cells were incubated for 48 h, 100 μL MTT solutions (2 mg/mL) were added to each well and the plate was incubated for another 4 h at 37 °C. The formed formazan crystals were dissolved in DMSO (100 μL/well) with constant shaking for 5 min. Absorbance of the solution was then measured with amicroplate reader at 490 nm. This assay was conducted in triplicate.

### Colony formation assays

Cells were seeded into 6-well culture plates (2 × 10^2^ cells/well). After 24 h, Cells were treated with different concentrations of AO (10, 20, 40 μM) and incubated for 48 h at 37 °C in 5% CO_2_ humidified environment. Then changed the medium and kept them growing for 10–12 days. Then clones were fixed with methanol and stained with Giemsa for 10 min. Stained clones which contain more than 50 cells were counted and cloning efficiency was calculated by the formula: cloning efficiency = (clone number/total cell number) × 100%. Furthermore, the cell survival fraction was counted and the cell survival curve was drawn.

### Small interfering RNA (siRNA)

RNAi (RNA interference) RNAi-mediated down-regulation of GOT1 was performed by one siRNA (small interfering RNA, 5′CUCUAACCCUGAGCUCUUU-3′) targeting GOT1 into SW1990 cells following the manufacturer’s instructions for Lipofectamine RNAi MAX reagent (Invitrogen). Another siRNA (5′CUCAAACGUUGAGAUCCUU-3′) was chosen as control. Knockdown efficiency was assessed by Western blotting at 48 h after induction of siRNA. Cell viability of GOT1 knockdown cells after treating with AO was measured by MTT assays.

### Cell proliferation assay

Cells were plated in 24-well plates at 2000 cells per well in 0.5 ml of media. To deprive OAA, cells were plated in complete culture media (10 mM glucose and 2 mM Gln), which was exchanged with medium supplemented with 10% FBS the following day. Media was not changed throughout the course of the experiment. At the indicated time points, cells were fixed in 10% formalin and stained with 0.1% crystal violet. Dye was extracted with 10% acetic acid and the relative proliferation was determined by optical density (OD) value at 595 nm.

### Cell cycle analysis

For determining phase distribution of DNA content, propidium iodide (PI) staining was performed. In brief, cells were respectively treated with vehicle or different concentrations of AO for 48 h. Floating and adherent cells were collected, washed in ice-cold phosphate-buffered saline (PBS) and fixed overnight in 70% ethanol at 4 °C. After centrifugation at 2500 rpm for 5 min at 4 °C, the cell pellet was stained with 30 μg/mL PI and 60 μg/mL RNase A in PBS buffer for 30 min inthe dark. Flow cytometric analyses were performed by using a FACSort flowcytometer provided with the Cell-Quest software (Becton–Dickinson, USA). Cell cycle assay was performed with three independent experiments.

### Annexin V apoptosis assay

Annexin V and PI staining was carried out using a Annexin V-FITC Apoptosis Detection Kit I (BD Pharmingen™, San Diego, CA). In brief, after treatment with different concentrations of AO for 48 h, SW1990 cells were washed twice with ice-cold PBS. After centrifugation at 1200 rpm for 5 min at 4 °C, cells were collected and adjusted to a density of 1 × 10^5^ cells/mL. Apoptotic cells were detected by fluorescent stainingas follows. Collected cells were incubated with Annexin V-FITC and propidium iodide (PI) in binding buffer for 15 min at room temperature in the dark, and stained cells were immediately subjected to flow cytometry analyses using a FACS Verse flow cytometer (Becton–Dickinson, USA). Apoptotic assay was performed with three independent experiments.

### Wound scratch assay

SW1990 cells were planked in 6-well plates with 5 × 10^5^ cells/well for 24 h. When cells density reached to 85%, a sterilized 10 μL micro pipette tip was applied to make three straight scratches per well. The plates were then washed with PBS to remove the floating cells before the AO (0–40 μΜ) added. The cell migration extent was imaged by a digital microscopy at 0 h. 24 h and 48 h after the scratches were made. The wound area was analyzed by Image J. The rate of wound healing = [(the wound width of 0 h – 48 h)/0 h wound width] × 100%.

### Quantification of metabolites

For steady state metabolomics analysis, SW1990 cells were grown to 50% confluence in growth media (DMEM, 2 mM Gln, 10 mM glucose, 5% FBS) on 10-cm dishes in biological quadruplicate. A complete media change was performed 2 h before metabolite collection. The abundance of Gln (BioVision, #K55), Asp (Abcam, #ab102512), OAA (Abcam, #ab83428) and malate (BioVision, #K637) was determined by using quantification kits, according the manufacturer’s instruction. Briefly, 20 mg cells were collected and homogenized in 0.5 mL of buffers provided (on ice). After centrifuged at 4 °C for 10 min at 13,000 g, the supernatants were deproteinized using 10 K spin column (BioVision), analyzed and compared to standard curves.

### Oxygen consumption rate (OCR) and extracellular acidification rate (ECAR)

OCR and ECAR were determined using the XF24 Extracellular Flux Analyzer (SeAOorse Bioscience). Briefly, 5 × 10^4^ SW1990 cells were seeded onto 24-well plates and incubated overnight. Then, cells were washed with SeAOorse buffer (DMEM with phenol red containing 25 mM glucose and 2 mM Gln). Cell Mito Stress Test Kit was used to measure cellular mitochondrial function, 175 μL of SeAOorse buffer plus 25 μL each of 1 μM oligomycin, 0.5 μM FCCP, and 1 μM rotenone was automatically injected to determine the OCR, according to the manufacturer’s instructions. The Glycolysis Stress Test Kit was used to measure the glycolytic capacity, 25 μL each of 10 mM glucose, 1 μM oligomycin, and 100 mM 2-deoxyglucose (2DG) were added to determine the ECAR, according to the manufacturer’s instructions. OCR and ECAR values were normalized to cell numbers. Data were from triplicated experiments and were plotted as mean ± S.D.

### In vivo tumor xenograft study

Animal care and experimental procedures were conducted in accordance with the Institutional Animal Ethical Committee, Huazhong University of Science and Technology, China. Male CB-17/scid mice (8 weeks old) were purchased from Huafukang Laboratory Animal Research Center Co., Ltd. (Beijing, China). Animals were fed a standard rat chow with free access to tap water and housed in a temperature and humidity-controlled room with a 12 h light-dark cycle. SW1990-luc cells (3 × 10^6^) were resuspended in 0.1 ml PBS and injected subcutaneously at the right flank of mice. When tumors reached an average diameter of 3 mm, the mice were randomly grouped into model control, 2.5 mg/kg and 5 mg/kg, and then treated intraperitoneal injection (i.p.) with AO or normal saline every day. Tumor size was measured once a week using vernier caliper and their volumes (mm^3^) were calculated according to a standard formula: width^2^ × length / 2. After treatment for 2 weeks, the mice were sacrificed by deep anesthesia, and metabolites of tumor tissues were also analyzed according the protocols above with little modification. All animal experiments were carried following the protocols approved by the Laboratory Animal Center of the Huazhong University of Science and Technology. All animal experiments were performed in accordance with the Guide for the Care and Use of Laboratory Animals of Huazhong University of Science and Technology and approved by the Ethics Committee.

### Statistical analysis

All data are expressed as the means ± SD from at least three independent experiments. SPSS 13.0 (SPSS, Inc., Chicago, IL, USA) was used for statistical analysis. Student’s unpaired t-test was performed to analyze individual group statistical comparisons. Multiple-group comparisons were evaluated by one-way ANOVA with post hoc testing. Values of *P* < 0.05 were considered to be statistically significant.

## Result

### AO inhibited GOT1 activity

**Fig. 1:**
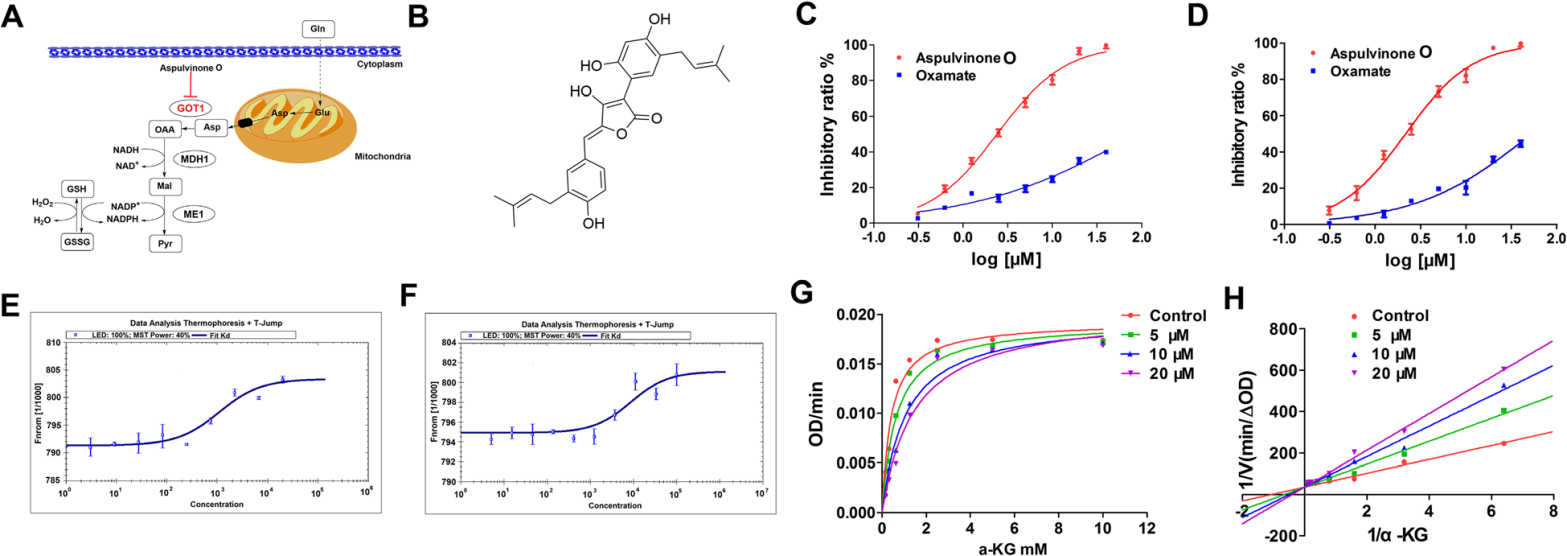
GOT1 is selectively targeted by AO. **a** Schematic depiction of the sequential reactions that convert Asp into pyruvate, catalysed by GOT1, MDH1, and ME1. **b** The chemical structures of AO. **c, d** AO selectively inhibited homo and murine GOT1 activity. **e, f** MST assay of the K_D_ value between AO and homo and murine GOT1. **g, h** The enzyme kinetics of AO on homo GOT1

Over 1500 compounds from the in-house Natural Products Library were selected for preliminary biological evaluation against GOT1. AO, which was isolated from rice culture of *Aspergillus terreus*, exhibited the best inhibitory effect against the activities of both purified homo and murine GOT1 with the IC_50_ values which were much lower than the selected positive control, oxamate (Fig. 1b-d).

Microscale thermophoresis (MST) was further employed to assay the binding affinity between AO and GOT1 proteins. This technology detects the fluorescent changes of molecules during thermophoresis to quantify protein-protein interactions or protein-small molecule interactions with high sensitivity and low analysis cost [22, 23]. The equilibrium dissociation constant (K_D_) values of AO with homo and murine GOT1 were 3.32 ± 1.18 μM and 5.42 ± 1.36 μM, respectively, which confirmed the specific binding to GOT1 (Fig. 1e-f). The enzyme kinetics of selected compound on GOT1 was then examined using purified homo GOT1. Lineweaver-Burk analyses indicated that the inhibitory effects were almost completely competitive with α-KG (Fig. 1g and h) [24].

### Molecular docking elucidated the binding mode of AO with GOT1

**Fig. 2:**
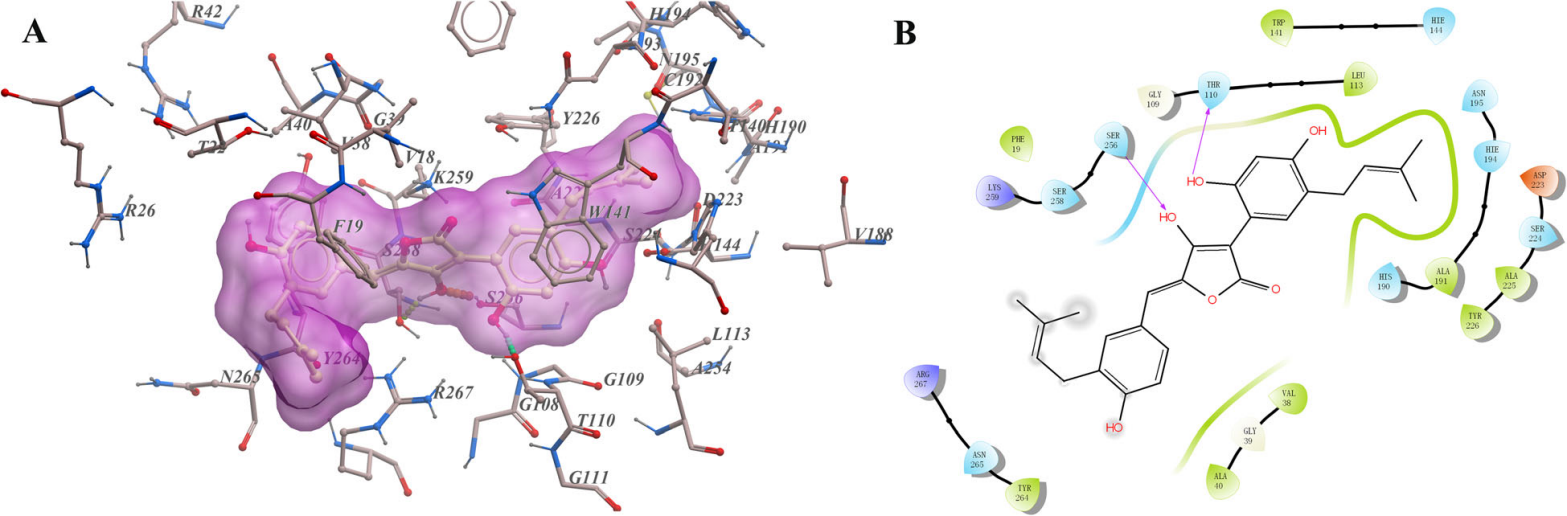
The low-energy binding conformations of AO bound to GOT1 generated by virtual docking. **a** Detailed view of AO binding in the active site of GOT1. **b** Ligand interaction diagram of AO with GOT1

In order to further elucidate the binding mode of AO with GOT1, molecular docking was carried out by using ICM 3.8.2 modeling software (MolSoft LLC, San Diego, CA) [25]. Ligand binding pocket residues were selected by using graphical. The lowest-energy binding conformation of AO with the enzyme was shown as Fig. 2. Docking results demonstrated that the compound bind to the active site of the enzyme. The binding pocket of AO was hand-gun shaped, where many hydrophobic amino acids, including Phe19, Leu113, Trp141, His144, His190, Ala225, Tyr226, and Tyr264, form a relatively hydrophobic envelop. AO formed obvious hydrophobic interactions with many of these amino acids. One benzene ring of AO also formed strong hydrophobic stacking with Trp141 (Fig. 2a). Hydrogen bonds were predicted between AO with Thr110 and Ser256 (Fig. 2b).

### AO inhibited GOT1 activity in pancreatic cancer cells

**Fig. 3:**
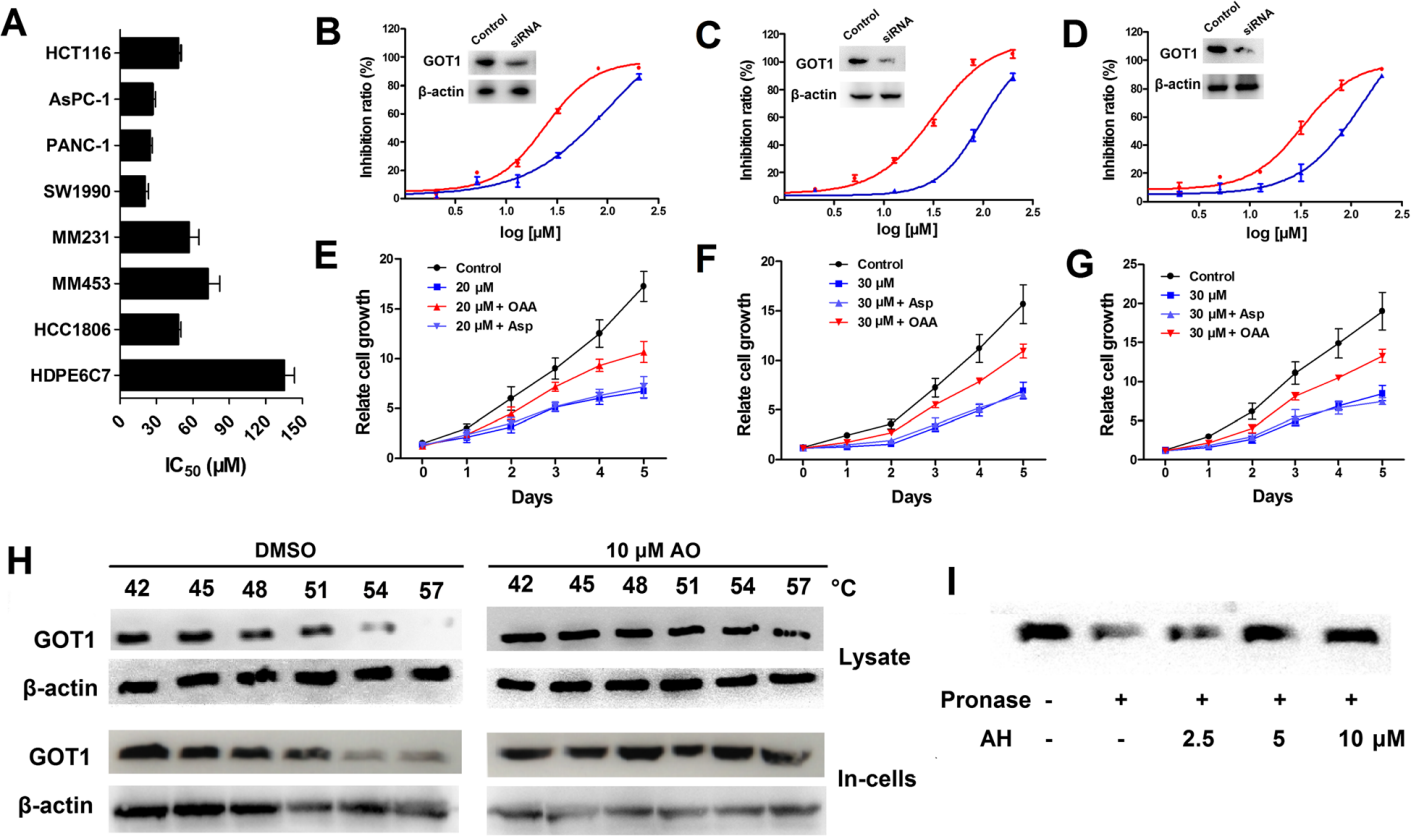
AO treatment specifically inhibits the proliferation of PDAC cells. **a** The IC_50_ values of AO for indicated cell lines. Cells were treated with AO at various concentrations for 48 h and processed for the MTT assay. **b**-**d** SW1990, AsPC-1 and PANC-1 cells were separately transfected with the scrambled siRNA (control) and GOT1-specific siRNA for 48 h, and the knockdown efficiency of GOT1 levels was analyzed by Western blotting. Then the cytotoxicity of AO on the GOT1 knockdown cell lines were markedly decreased compared with corresponding control cell lines. **e**-**g** SW1990, AsPC-1, PANC-1 cells were pretreated with OAA, Asp at indicated concentrations for 2 h, and then incubated with indicated concentrations of AO for 5 days. The cell number was determined by MTT assay. **h** A CETSA was performed to evaluate the interaction between AO and GOT1 a in SW1990 intact cells and cell lysates. **i** DARTS assay was performed on the SW1990 cell lysates

To explore the anti-tumor potential of AO against pancreatic cancer, we chose several human cancer cell lines, including breast (MM231, MM453, HCC1806), colorectal (HCT116), pancreas (PANC-1, AsPC-1, SW1990), along with nonmalignant human hepatic cell line (HDPE6C7) to test the growth-inhibitory effect of AO. As shown in Fig. 3a, after treatment for 24 h, AO exerted significantly greater cytotoxicity effects on PANC-1, AsPC-1 and SW1990 cell lines than on the other cell lines with IC_50_ values ranging from 20.54 to 26.80 μM. Furthermore, AO exerted minimal effects on the viability of the normal cell lines HPDE6-C7 (IC_50_ > 100 μM). These data indicated that AH shows selective antiproliferative effects and cytotoxicity against PDAC cells.

To assess whether the effects of AO are GOT1 dependent, we used siRNAs to knock down GOT1 expression. GOT1 protein levels were dramatic reduced in the pancreas cell lines treated with siRNA, which attenuated the sensitivity of the cells to AO (Fig. 3b-d). These results indicated that GOT1 levels influence cell sensitivity to AO. GOT1 converts Asp into OAA that eventually gets converted into pyruvate by a reaction catalyzed by malic enzyme 1 (ME1). Hence, OAA could re-establish the anaplerotic Gln metabolism of knockdown cells [26]. To further elucidate the contribution of GOT1 inhibition to AO-induced cell growth inhibition, we investigated whether the resultant OAA could antagonize AO. For this, SW1990, ASPC-1 and PANC-1 cells were pretreated with Asp or OAA for 12 h, and then exposed to AO (20 or 30 μM) for 5 days. Our results indicated that OAA (2 mM) could enhance cell growth under AO treatment conditions, while Asp barely attenuated the inhibitory effect of AO on cell viability (Fig. 3e-g).

To evaluate the in vivo interaction between AO and GOT1, we conducted a cellular thermal shift assay (CETSA) analysis [27, 28]. The CETSA is used for assessing target engagement by drugs in vivo based on the biophysical principle of the ligand-induced thermal stabilization of target proteins, and it will likely become a valuable tool for validating and optimizing drug target engagement. As shown in Fig. 3h, we found that AO treatment efficiently protected GOT1 protein from temperature-dependent degradation. Under DMSO control treatment, 50% of the GOT1 was degraded at 51 °C, whereas in the AO–treated samples, no obvious degradation was observed at temperatures of 57 °C in both intact cells and cell lysate. Meanwhile, drug affinity responsive target stability (DARTS), a relatively quick and straightforward approach to identify potential protein targets for small molecules that relies on the protection against proteolysis conferred on the target protein by interaction with a small molecule [29], was used to further monitor target engagement based on AO-induced stabilization of GOT1, as shown in Fig. 3i, AO could effectively mitigate pronase induced GOT1 proteolysis with a dose-dependent manner. Taken together, these results strongly indicate that AO could directly interact with GOT1, inhibit the enzymatic activity of GOT1 and selectively suppress the proliferation of PDAC cells.

### AO modulates Gln metabolism and ROS response

**Fig. 4:**
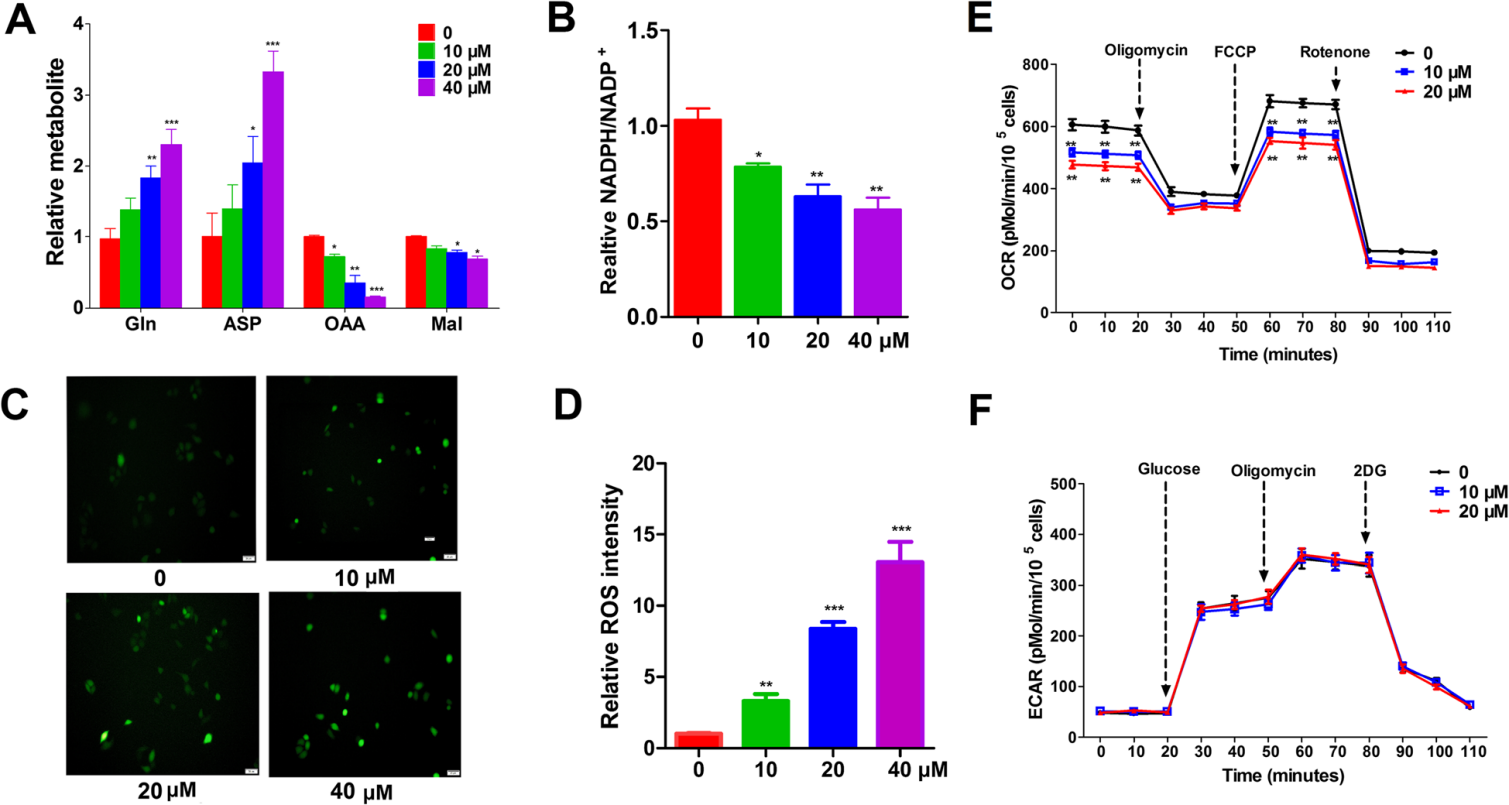
AO treatment induced Gln metabolism and modulates mitochondrial respiration. **a** Relative metabolite abundance in SW1990 cells treated with 10, 20, 40 μM AO. **b** NADP 1/NADPH ratio in in SW1990 cells treated with 10, 20, 40 μM AO. **c, d** Measurement of intracellular ROS using carboxyH2DCFDA in control and AO treatments. **e, f** OCR and ECAR analysis of SW1990 cells treated with 10 and 20 μM AO. *P < 0.05, **P < 0.01, ***P < 0.001 compared with the vehicle control

In addition, GOT1 is involved in Gln-dependent NADPH production of pancreatic cancer. GOT1 catalyses the reversible conversion of Asp to OAA. To examine the influence of AO in Gln metabolism, we detected the associated metabolites. OAA and malate were decreased by AO treatment, with corresponding accumulation of Asp. Consequently, cells treated with AO showed remarkably decreased NADPH/NADP^+^ ratio (Fig. 4a, b). Next, we investigated the physiological relevance of AO treatment with ROS response. ROS would induce pathology due to damages in DNA, proteins, and lipids at high levels. As a result, oxidative damage may cause senescence, growth inhibition, and cell death. We measured ROS levels using 2,7-dichlorofluorescin diacetate (DCFDA) and found that AO induced an increase of cellular ROS in SW1990 cells. We concluded that cells generated increased ROS due to inhibited GOT1. All these results suggest that the inhibitory effect of AO could block the Gln-dependent NADPH production and is involved in the redox homeostasis of PDAC cells (Fig. 4c, d).

GOT1 participates in malate-aspartate shuttles that coordinate glycolysis and mitochondria respiration. To define the influence of a GOT1 inhibitor on metabolic fluxes, we determined the extracellular acidification rate (ECAR), an indicator of lactate production and glycolysis, and the oxygen consumption rate (OCR), an index of mitochondrial respiration [30, 31]. AO treatment reduced OCR and induced no obvious influence on ECAR. These data suggested that GOT1 inhibition could suppress malate-Asp shuttling and mitochondrial respiration. (Fig. 4e, f).

### AO induced apoptosis in pancreatic cancer cells

**Fig. 5:**
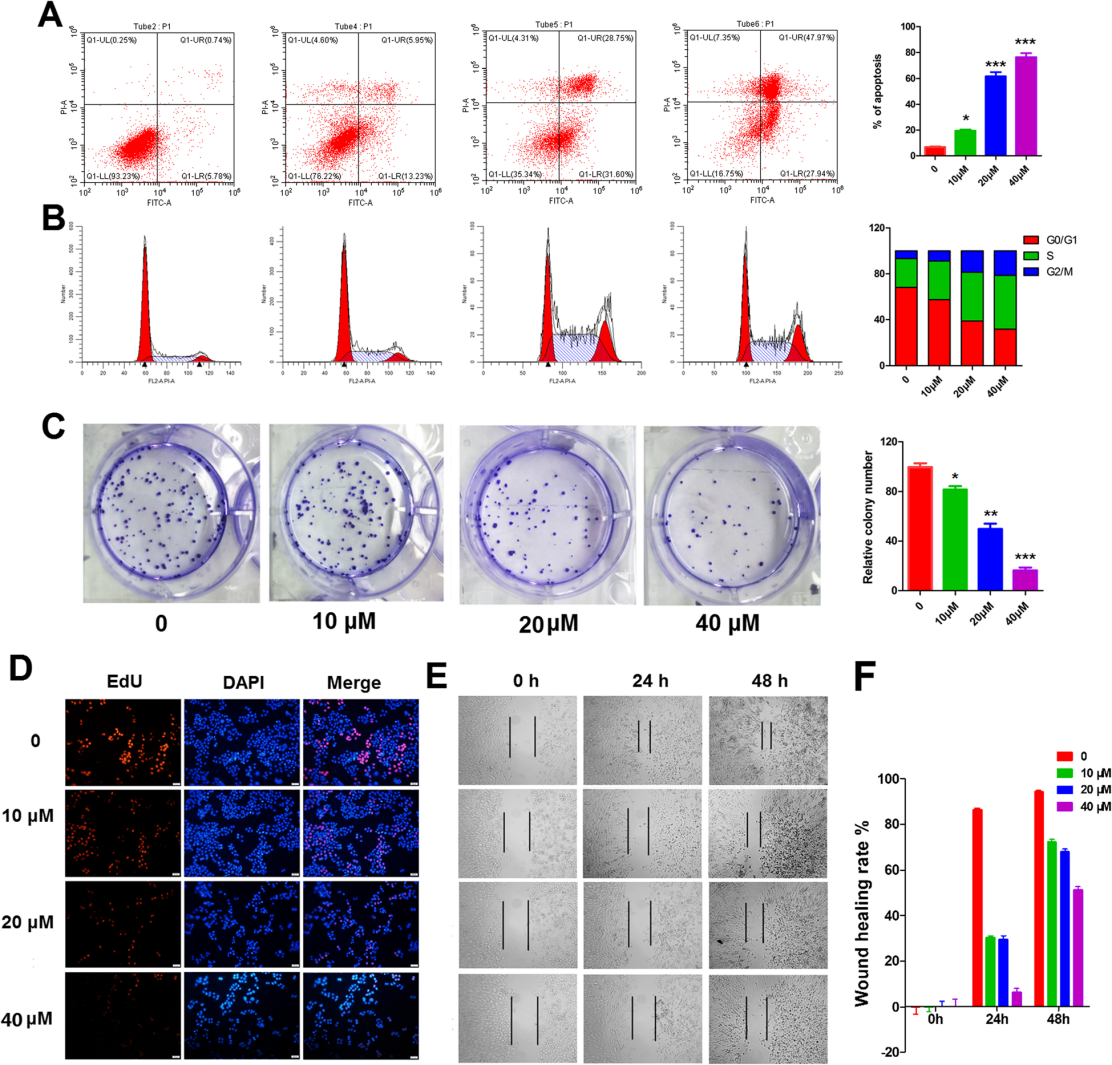
AO induces apoptosis, cell cycle arrest and MMP loss in SW1990 cells in vitro. **a** SW1990 cells were treated with AO for 48 h, and the apoptotic cells were evaluated and quantified by Annexin V/PI staining. **b** Cell cycle analysis of SW1990 cells in control and AO treatment. **c** 14-day Colony formation assays for cells cultured in control and AO treatments. **d** DAPI and EdU double staining was used to determine the effect of different concentrations of AO on the proliferation of SW1990 cells. **e, f** Scratch wound healing of SW1990 cells was evaluated using cell scratch wound healing assay. Images from the same area were captured at time 0 h, 24 h and 48 h after wound infliction. The mean ± SD of three experiments is shown. *P < 0.05, **P < 0.01, ***P < 0.001 compared with the vehicle control

Apart from inhibiting of proliferation, we also explored the mechanisms of AO-induced growth inhibition by flow cytometry. Annexin-V propidium iodide double staining (Annexin-V/PI staining) revealed that AO at 10, 20 and 40 μM induced 11.4, 61.7 and 76.4% apoptotic death compared with 4.8% in SW1990 cells (Fig. 5a). At the same time, we examined the cell cycle distributions of SW1990 cells treated with increasing doses of AO for 48 h. Flow cytometric analysis demonstrated that AO induced cell cycle arrest at the S phase in SW1990 cells. As shown in Fig. 5b, there was an increase in the fraction of SW1990 cells in the S phase (33.6, 42.5, 46.8%, compared to 25.1% in untreated cells) and a decrease in the percentage of cells in the G0/G1 phase (57.6, 38.9, 31.8%, compared to 68.1% in untreated cells). Meanwhile, the G2 phase also showed relatively low growth with 8.8, 18.7, 21.3% comparing to 6.7% of control cells).

To determine if reduced growth in AO treatment is due to reduced clonogenicity we conducted colony formation assays and identified that cells treated with serious concentrations of AO had reduced clonogenicity compared to the cells that are cultured in control (Fig. 5c). Subsequently, the EdU staining analysis and wound healing cell migration test further demonstrated the anti-proliferation effect of AO against SW1990 cells (Fig. 5d, e, f). All data above indicated cell cycle arrest in the G1/S transition in PDAC cells with AO treatment that results in reduced rate of cell cycle progression and growth.

### In vivo antitumor efficacy of AO in a xenograft mouse model

**Fig. 6:**
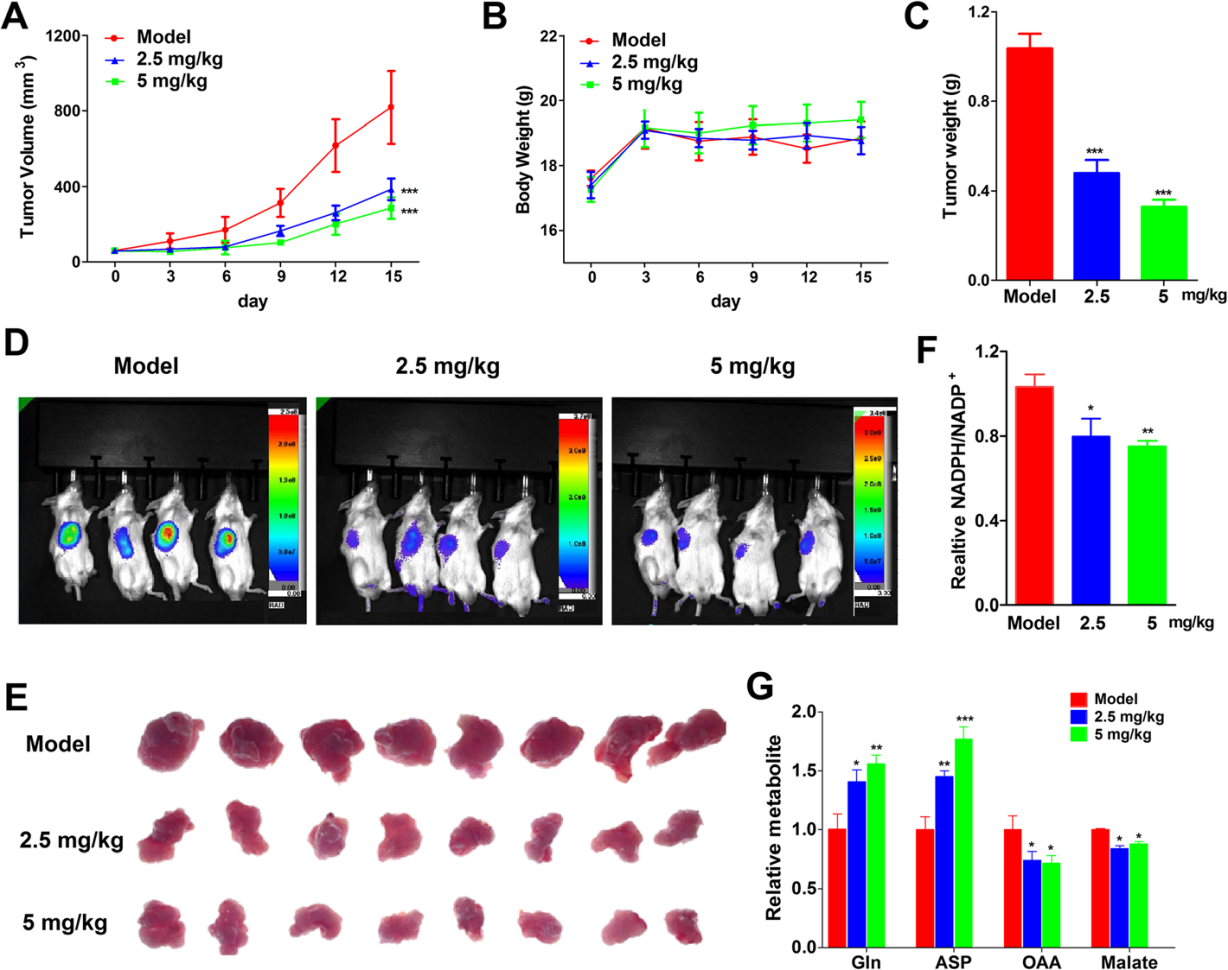
AO suppresses SW1990 tumor development in vivo. Tumor growth curve (**a**), mouse weight (**b**), and tumor weight (**c**) for SW1990 xenografts in CB-17/scid mice treated with 2.5 or 5 mg/kg/day AO or an equal volume of vehicle for 14 consecutive days. **d** Bioluminescence imaging of tumor at day 7. **e** Tumor tissues of xenograft mice after 14 days treatment. **f** NADPH/NADPH^+^ ratio in in tumor tissues from different groups. **g** Relative metabolite abundance in tumor tissues from different groups. The differences among groups were compared by analysis of variance (ANOVA). All data were presented as mean ± SD. *P < 0.05, **P < 0.01, ***P < 0.001 compared with the vehicle control

We next evaluated the in vivo anti-tumor efficacy of AO. In the xenograft model, SW1990-luc cells were inoculated subcutaneously into the left flank of each CB-17/scid mouse to develop xenograft models. Seven days later, the mice were then treated by intraperitoneal injection with vehicle or AO (2.5 and 5 mg/kg/d) for 14 days. We found that treatment with AO could significantly inhibit the growth of SW1990 xenografts and reduce the weights of tumors compared with the vehicle-treated group (Fig. 6a-e). To evaluate the effect of AO on tumor tissue metabolism, OAA and malmate were also decreased by AO treatment, with corresponding accumulation of Gln and Asp. Consequently, NADPH/NADP^+^ ratio was remarkably declined in the same manner with assays in vivo (Fig. 6f, g). Consequently, the NADPH/NADP^+^ ratio decreased substantially, similar to the results of the in vitro assays. In addition, no remarkable loss of body weight and changes of histomorphology were observed in treated or vehicle-treated mice, which indicated that AO was a safe and effective antitumour agent that could be a potent GOT1 inhibitor.

## Discussion

Malignant cells exhibit metabolic changes, compared to their normal counterparts, owing to both genetic and epigenetic alterations. Metabolic reprogramming allows cancer cells to sustain uncontrolled proliferation by rapid generation of ATP, biosynthesis of macromolecules, and maintenance of redox status [32, 33]. Furthermore, it is only during the past decade that targeting cancer metabolism has emerged as a promising strategy for the development of selective antineoplastic agents. The characteristic metabolic phenotype seen in cancer cells is the Warburg effect, the tendency of malignant cells to metabolize glucose via aerobic glycolysishigh glucose uptake. Addition to glucose, reprogramming of Gln metabolism enables efficient macromolecular biosynthesis in proliferating cells. PDAC cells utilize Gln metabolism to increase the NADPH/NADP^+^ ratio, coupling cellular redox status with this unique metabolic pathway [34].

PDAC is a lethal cancer type that is projected to become the second leading cause of cancer-related deaths in the United States by 2020 and, at present, has a 5-year survival rate of 6% [35, 36]. Recently, considerable literature have demonstrated that a KRAS regulated non-canonical Gln metabolism pathway that enables proliferation and tumor growth in PDAC cell lines. Specifically, KRAS dependent upregulation of GOT1 in PDAC results in Gln-derived aspartate being converted to OAA by GOT1 in the cytoplasm, which is ultimately lead to the generation of NADPH and further maintain redox balance [15]. Thus, GOT1 inhibitors may provide a much-needed therapeutic approach for selectively targeting PDAC.

Over 1500 compounds from the in-house NP library were screened against the GOT1 enzymatic activity. Several compounds exhibiting various degrees of inhibitory activity, in which, AO as the best performed compound was selected for further biological evaluation. AO, a lignose with a highly functionalized, was found to have the best inhibitory activity against both homo and murine GOT1 with a completely competitive manner. From the generated docking model, AO was extending into the substrate binding pocket of the enzyme. MST assays indicating the strong affinity between AO and recombinant GOT1 protein, as well as the competitive inhibition of AO for the enzymatic activity, were all consistent with the virtual docking hypothesis.

Then we demonstrated that AO can selectively inhibit pancreatic cancer cell proliferation, and downregulation of GOT1 in pancreatic cancer cells significantly enhanced AO-induced growth-inhibitory effects. Association with the CETSA and DARST analysis, these results indicated that AO tightly binds with GOT1 to form a complex to inhibit GOT1 activity in intracellular. Previous studies reveal the role of non-canonical anaplerotic Gln metabolism in the generation of NADPH and possibly ROS regulation through coupling with other redox balance pathways [37]. In our studies, the increase in ROS levels is primarily due to the upregulation in the transcription of NADPH oxidases during AO treatment. We then further confirmed AO can block the cell cycle, induce cell apoptosis, decreased colony formation and inhibited a SW1990 cell-induced xenograft model, all of which indicated the suppressive effect of AO on pancreatic cancer growth both in vitro and in vivo.

## Conclusions

In conclusion, our findings indicate that the novel compound AO, as a selective GOT1 inhibitor, could target the Gln metabolism activity in PDAC cells and suppress the cells growth both in vitro and in vivo. The potential therapeutic value of AO can be explored further to develop novel anti-tumor agents.

## Additional file

Additional file 1: **Figure S1.** 1H NMR Spectrum of Aspulvinone O in MeOH-*d*_4_. **Figure S2.** ^13^C NMR Spectrum of Aspulvinone O in MeOH-*d*_4_. **Figure S3.** Original Western blot images. (DOCX 1258 kb).

## Data Availability

The dataset supporting the conclusions of this article is included within the article and its additional file.

## Abbreviations

AO: Aspulvinone O
Asp: Aspartate
CETSA: Cellular thermal shift assay
DARTS: Drug affinity responsive target stability
ECAR: Extracellular Acidification Rate
Gln: Glutamine
Glu: Glutamate
GOT1: Aspartate transaminase
MDH1: Malate dehydrogenase 1
ME1: Malic enzyme 1
MST: Microscale Thermophoresis
OAA: Oxaloacetate
OCR: Oxygen Consumption Rate
PDAC: Pancreatic ductal adenocarcinoma
ROS: Reactive oxygen species
siRNA: Small interfering RNA
TCA: Tricarboxylic acid
α-KG: Alpha-ketoglutarate
