# Supplementary material for: Aspulvinone O, a natural inhibitor of GOT1 suppresses pancreatic ductal adenocarcinoma cells growth by interfering glutamine metabolism
Source: Cell Commun Signal. 2019 Aug 30;17:111. doi: 10.1186/s12964-019-0425-4 (PMC6717386; doi:10.1186/s12964-019-0425-4)

**Electronic Supporting Information(ESI)**

**Aspulvinone O, a natural Inhibitor of GOT1 suppresses pancreatic ductal adenocarcinoma cells growth by interfering glutamine metabolism**

Weiguang Sun ^a,1^, Shanshan Luan ^a,1^, Changxing Qi ^a,1^, Qingyi Tong ^a^, Shan Yan ^a^, Hua Li ^a,b*^, Yonghui Zhang ^a*^

^1^Hubei Key Laboratory of Natural Medicinal Chemistry and Resource Evaluation, School of Pharmacy, Tongji Medical College, Huazhong University of Science and Technology, Wuhan, 430030, China.

^2^ Wuya College of Innovation, Key Laboratory of Structure-Based Drug Design & Discovery, Ministry of Education, Shenyang Pharmaceutical University, Shenyang, 110016, China.

*Correspondence and requests for materials should be addressed to Hua Li (email: [li_hua@hust.edu.cn](mailto:li_hua@hust.edu.cn)), Yonghui Zhang (email: zhangyh@mails.tjmu.edu.cn)

^†^These authors contributed equally to this work.

**Isolation of Aspulvinone O**

The *Aspergillus terreus* strain was isolated from the soil collected from the bottom of Yangzi River at Wuhan, China. The DNA sequence data for this strain have been deposited in DDBJ/EMBL/GenBank under the accession number KT360948. The strain of *Aspergillus terreus* was cultured on potato dextrose agar (PDA) at 28 °C for 7 days to prepare the seed culture. Cut the PDA into small pieces (approximately 0.6×0.6×0.6 cm^3^) into 50 Erlenmeyer flasks (5 L) which had previously sterilized by autoclaving and each flasks containing 1000 mL distilled water and 1000 g rice. After the incubation, we used ethanol to stop the growth of fungus and then extracted with ethanol. The reduced pressure distillation was used to remove the ethanol and yield 880.0 g brown extract. Furthermore, the EtOH extract of this culture was chromatographed by a silica gel chromatography column (CC) eluted with CH_2_Cl_2_/MeOH (10:1–1:1), and six fractions (Fr. 1–Fr. 6) were progressively obtained. We separated the Fr.5 with Sephadex LH-20 CC (CHCl_3_-MeOH, 1:1) to yield four subfractions (Fr. 4.1–Fr. 4.4), and then the subfraction Fr. 4.2 was subjected to a chromatographed on ODS (MeOH-H_2_O, 20%–80%) to yield nine mixtures (A–I). Mixture E was purified by repeated semi-preparative HPLC to yield aspulvinone I (331 mg).

**Supplementary Figure 1. 1H NMR Spectrum of Aspulvinone O in MeOH-*d*_4_**


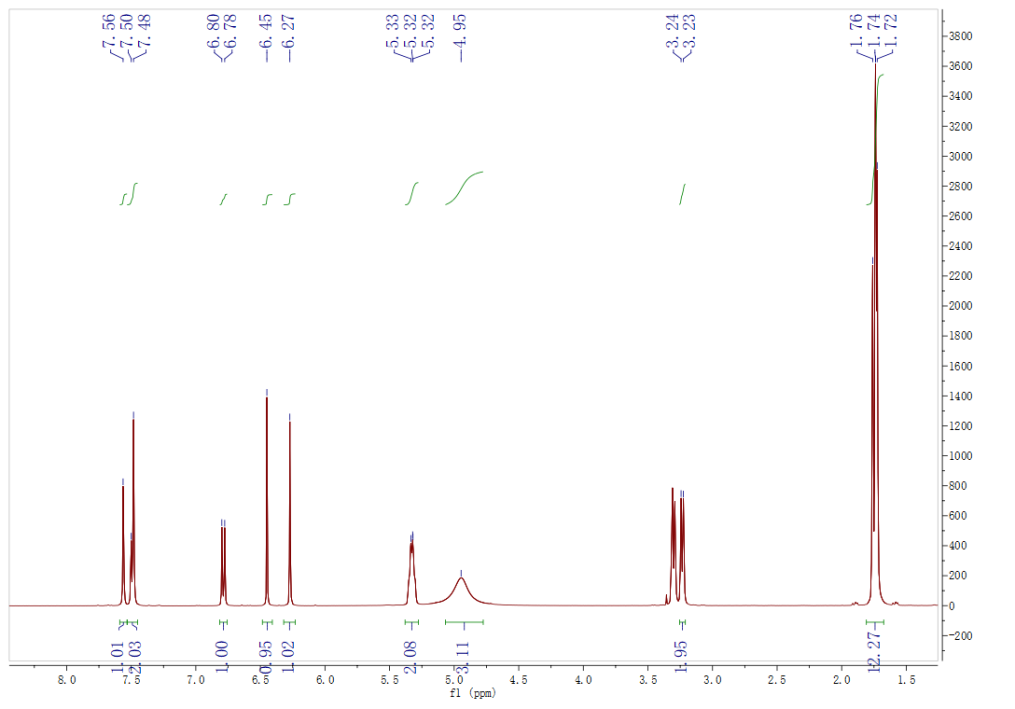


**Supplementary Figure 2. ^13^C NMR Spectrum of Aspulvinone O in MeOH-*d*_4_**


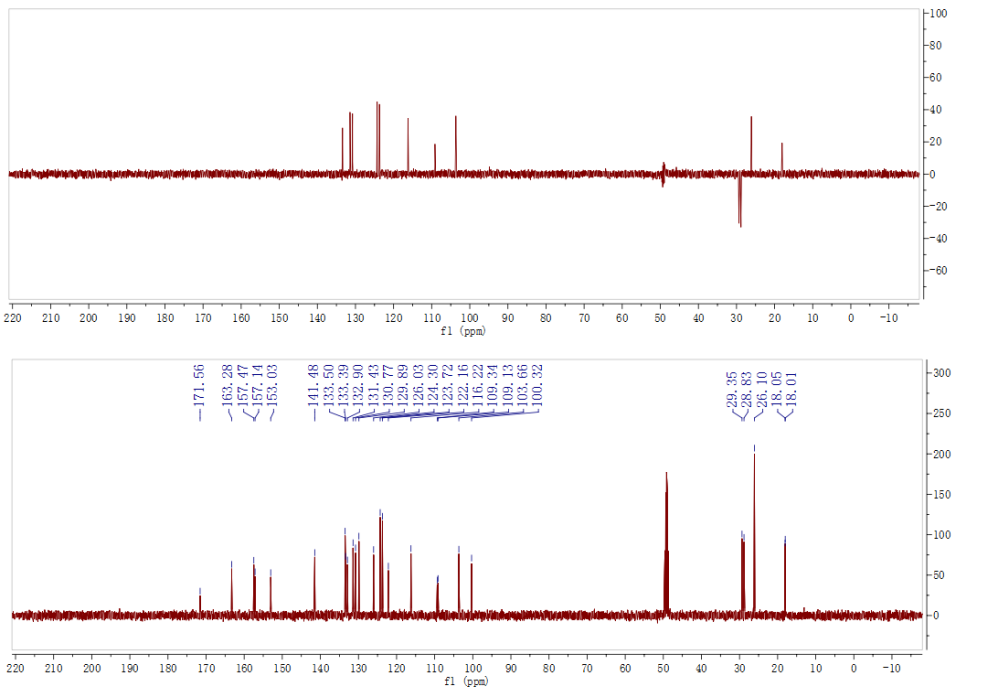


**Supplementary Figure 3. Original Western blot images**


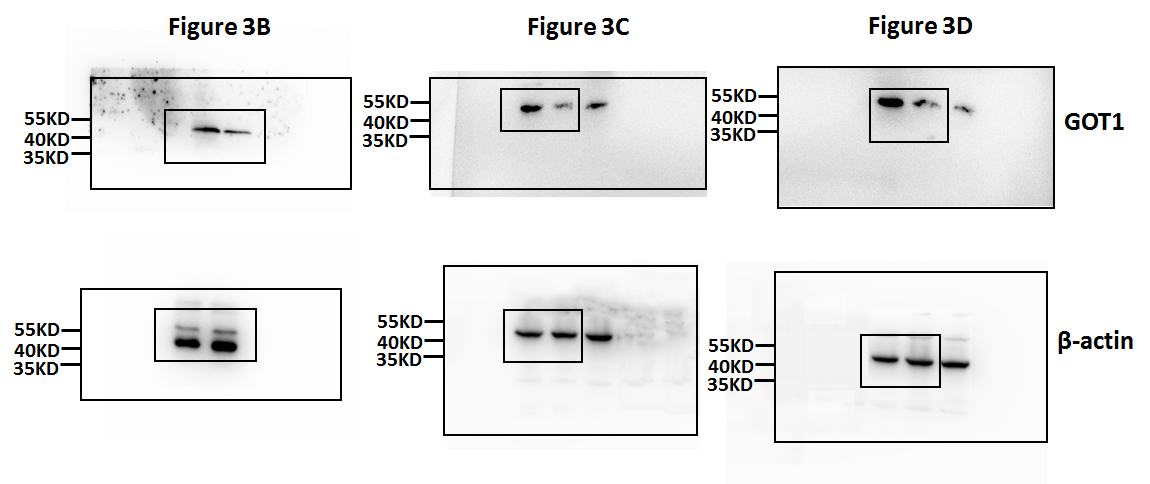


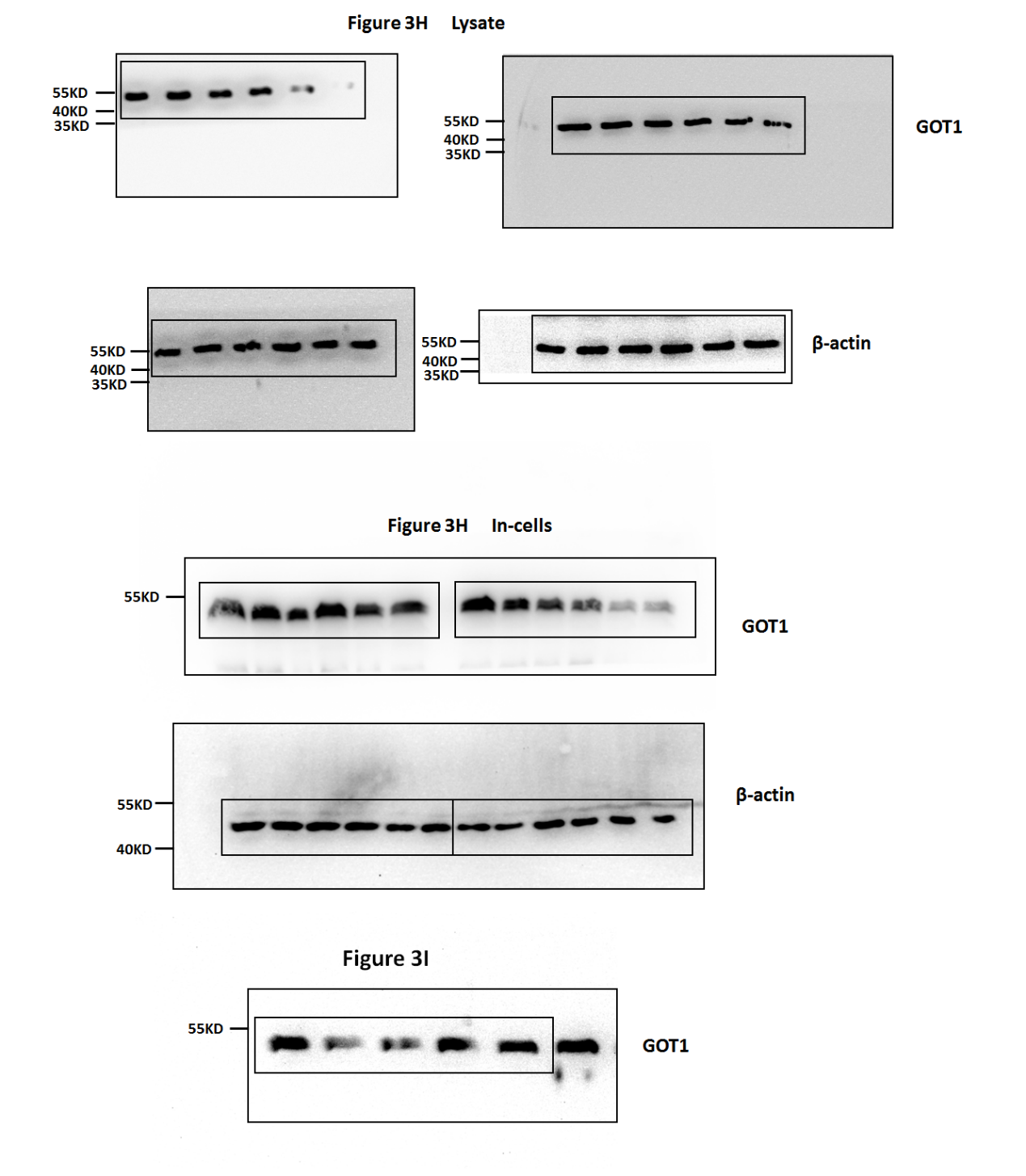

Supplement: Supplementary file 1 — Figure S1. 1H NMR Spectrum of Aspulvinone O in MeOH-d4. Figure S2. 13C NMR Spectrum of Aspulvinone O in MeOH-d4. Figure S3. Original Western blot images. (DOCX 1258 kb). [file 12964_2019_425_MOESM1_ESM.docx]
